# Proanthocyanidin Characterization, Antioxidant and Cytotoxic Activities of Three Plants Commonly Used in Traditional Medicine in Costa Rica: *Petiveria alliaceae* L., *Phyllanthus niruri* L. and *Senna reticulata* Willd.

**DOI:** 10.3390/plants6040050

**Published:** 2017-10-19

**Authors:** Mirtha Navarro, Ileana Moreira, Elizabeth Arnaez, Silvia Quesada, Gabriela Azofeifa, Diego Alvarado, Maria J. Monagas

**Affiliations:** 1 Department of Chemistry, University of Costa Rica (UCR), Rodrigo Facio Campus, San Pedro Montes Oca, San Jose 2060, Costa Rica; mnavarro@codeti.org; 2 Department of Biology, Technological University of Costa Rica (TEC), Cartago 7050, Costa Rica; imoreira@itcr.ac.cr (I.M.); earnaez@itcr.ac.cr (E.A.); 3 Department of Biochemistry, School of Medicine, University of Costa Rica (UCR), Rodrigo Facio Campus, San Pedro Montes Oca, San Jose 2060, Costa Rica; silvia.quesada@ucr.ac.cr (S.Q.); gabriela.azofeifacordero@ucr.ac.cr (G.A.); 4 Department of Biology, University of Costa Rica (UCR), Rodrigo Facio Campus, San Pedro Montes Oca, San Jose 2060, Costa Rica; luis.alvaradocorella@ucr.ac.cr; 5 Institute of Food Science Research (CIAL), Spanish National Research Council (CSIC-UAM), C/Nicolas Cabrera 9, 28049 Madrid, Spain

**Keywords:** *P. alliaceae*, *P. niruri*, *S. reticulata*, UPLC, TQ-ESI-MS, proanthocyanidins, mass spectrometry, antioxidant, cytotoxicity

## Abstract

The phenolic composition of aerial parts from *Petiveria alliaceae* L., *Phyllanthus niruri* L. and *Senna reticulata* Willd., species commonly used in Costa Rica as traditional medicines, was studied using UPLC-ESI-TQ-MS on enriched-phenolic extracts. Comparatively, higher values of total phenolic content (TPC), as measured by the Folin-Ciocalteau method, were observed for *P. niruri* extracts (328.8 gallic acid equivalents/g) than for *S. reticulata* (79.30 gallic acid equivalents/g) whereas *P. alliaceae* extract showed the lowest value (13.45 gallic acid equivalents/g). A total of 20 phenolic acids and proanthocyanidins were identified in the extracts, including hydroxybenzoic acids (benzoic, 4-hydroxybenzoic, gallic, prochatechuic, salicylic, syringic and vanillic acids); hydroxycinnamic acids (caffeic, ferulic, and p-coumaric acids); and flavan-3-ols monomers [(+)-catechin and (−)-epicatechin)]. Regarding proanthocyanidin oligomers, five procyanidin dimers (B1, B2, B3, B4, and B5) and one trimer (T2) are reported for the first time in *P. niruri*, as well as two propelargonidin dimers in *S. reticulata*. Additionally, *P. niruri* showed the highest antioxidant DPPH and ORAC values (IC_50_ of 6.4 μg/mL and 6.5 mmol TE/g respectively), followed by *S. reticulata* (IC_50_ of 72.9 μg/mL and 2.68 mmol TE/g respectively) and *P. alliaceae* extract (IC_50_ >1000 μg/mL and 1.32 mmol TE/g respectively). Finally, cytotoxicity and selectivity on gastric AGS and colon SW20 adenocarcinoma cell lines were evaluated and the best values were also found for *P. niruri* (SI = 2.8), followed by *S. reticulata* (SI = 2.5). Therefore, these results suggest that extracts containing higher proanthocyanidin content also show higher bioactivities. Significant positive correlation was found between TPC and ORAC (*R*^2^ = 0.996) as well as between phenolic content as measured by UPLC-DAD and ORAC (*R*^2^ = 0.990). These findings show evidence for the first time of the diversity of phenolic acids in *P. alliaceae* and *S. reticulata*, and the presence of proanthocyanidins as minor components in latter species. Of particular relevance is the occurrence of proanthocyanidin oligomers in phenolic extracts from *P. niruri* and their potential bioactivity.

## 1. Introduction

Characterization and quantification of secondary metabolites and their bioactivity studies are essential to increase the knowledge on plants with traditional medicinal uses towards efficient and safe utilization. Three plant species of special relevance because of their widespread utilization in traditional medicine in Costa Rica as anti-inflammatories are *Petiveria alliaceae*, *Phyllanthus niruri* and *Senna reticulata* [1]. *Senna reticulata* is a shrub belonging to the *Fabaceae* family, originally from Mesoamerica and South America (to Brazil), whose leaves and stems are used traditionally for rheumatism and skin conditions [2]. *Phyllanthus niruri* is a shrub native to America and tropical areas of India and China, which belongs to the *Phyllanthaceae* family, and has diuretic and hepatoprotective properties [3]. Finally, *Petiveria alliacea*, which belongs to the *Phytholacaceae* family, is distributed from the South of the United States to Brazil and its traditional uses include analgesic, anticoagulant and hypoglycemic properties [4].

Studies have attributed anti-inflammatory and other bioactivities mainly to anthraquinones present in *S. reticulate* [2], sulfur containing compounds in *P. alliaceae* [4], and lignans such as phyllanthosides in the case of *P. niruri*. More recent studies have shown the synergic effect of different families of phenolic compounds in bioactivity studies, for instance showing the important effects of proanthocyanidins on antioxidant and cytotoxic bioactivities [5,6]. Regarding phenolic compounds, mainly flavonoids such as kaempferol derivatives have been reported in *S. reticulata* [2] [7]; quercetin derivatives in *P. alliacea* [8] and *P. niruri* [9]; whereas caffeic acid derivatives and ellagitannins have also been reported in *P. niruri* [10]. Flavan-3-ols, including catechin and epicatechin monomers have been reported in *P. niruri* [11], whereas no detailed studies on proanthocyanidin oligomers have been performed in any of these three species.

Proanthocyanidins are condensed flavan-3-ols that constitute an important group of polyphenols because of their bioactivities, among others, ant-inflammatory, antioxidant and anti-cancer activities [12]. Despite the increasing number of studies on phenolics, the characterization of proanthocyanidins remains a complex task because of the need for high-end techniques such as High-Resolution Mass Spectrometry (HRMS). On the other hand, it has been argued that these bioactivities could be mediated by redox interaction, since the regulation on redox homeostasis has been implicated in the control of the transition from cell proliferation to cell differentiation and cell cycle progression in both plants and animals [13]. However, the mechanisms and factors that could affect these bioactivities remain to be elucidated [14], suggesting the importance of these studies.

Since proanthocyanidin composition of *P. alliaceae*, *P. niruri* and *S. reticulata* have been scarcely studied and because of findings demonstrating the synergic contribution of proanthocyanidins on plants whose bioactivities were attributed solely to other metabolites [5,15], the objective of this work was to obtain phenolic extracts from these three plant species and to characterize them UPLC-DAD-ESI-TQ-MS. Evaluation of the antioxidant activity through DPPH and ORAC methods, as well as the assessment of cytotoxicity in AGS adenocarcinoma gastric cells, SW620 adenocarcinoma colon cells, and Vero normal cells, was also carried out in the different extracts.

## 2. Results and Discussion

### 2.1. Phenolic Yield and Total Phenolic Contents

The extraction process described in the Materials and Methods section, allowed the phenolic enriched fractions to be obtained, as summarized in Table 1. *S. reticulata* presented the highest yield (6.53%) whereas *P. alliaceae* showed the lowest value (5.03%). The total phenolic contents (TPC) shown in Table 1, also resulted in comparatively lower values for *P. alliaceae* extract (13.45 gallic acid equivalents/g dry extract) than *P. niruri* extract (328.80 gallic acid equivalents/g dry extract), which exhibited the highest values. These results agree with few reports indicating lower TPC values for an hydroalcoholic extract of *P. alliaceae* [16], and for an aqueous extract of *S. reticulata* [17]. However, higher TPC values, in the range of 263–270 gallic acid equivalents/g, which are slightly lower than our findings have been reported for ethanolic extracts of *P. niruri* [10,18]. Table 1 also summarizes the total proanthocyanidin (PRO) content for the different extracts. *P. niruri* showed the highest PRO content (322.23 cyanidin chloride equivalents/g dry extract) whereas no content was found in *P. alliaceae. S. reticulata* showed intermediate values for both TP and PRO contents. Thus, phenolic content varied according to plant species, the highest values for both TPC and PRO clearly corresponding to *P. niruri*.

### 2.2. Phenolic Profile by UPLC-DAD-ESI-TQ-MS Analysis

**Figure 1:**
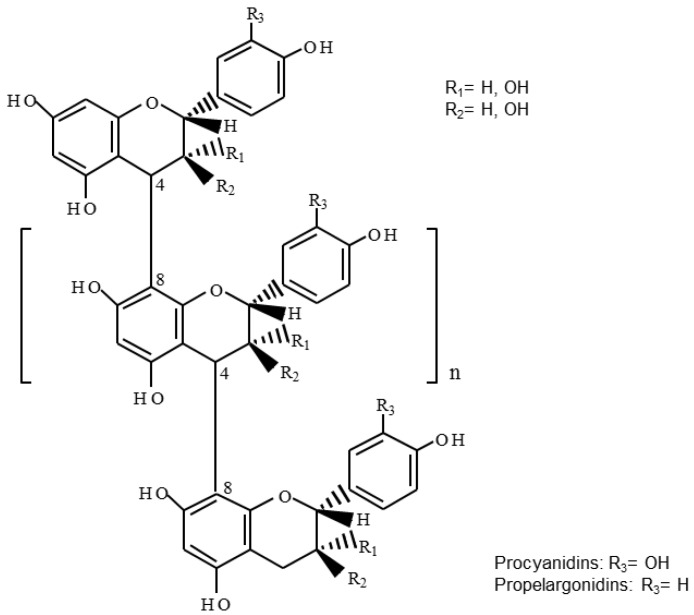
General chemical structure of B-type proanthocyanidins: procyanidins (composed by (epi) catechin units) and propelargonidins (composed by (epi) afzelechin units).

Table 2 summarizes the results of UPLC-DAD-ESI-TQ-MS analysis performed in the different extracts, as described in the Materials and Methods section. Also, Figure S1 and Table S1 (Supplementary Materials) show base chromatograms and main MS/MS parameters respectively. Among the 28 phenolic compounds screened, a total of 20 phenolic compounds were characterized and quantified, (benzoic, 4-hydroxybenzoic, gallic, protocatechuic, salicylic, syringic and vanillic acids); hydroxycinnamic acids (caffeic, ferulic, and p-coumaric acids); and flavan-3-ols monomers [(+)-catechin and (−)-epicatechin)], procyanidin dimers, propelargonidin dimers and procyanidin trimers). To our knowledge, our findings report for the first time the presence of proanthocyanidin oligomers (Figure 1) in extracts from *P. niruri* and *S. reticulata*.

In fact, five different procyanidin dimers, namely B1, B2, B3, B4 and B5 were found in *P. niruri* extract, two propelargonidin dimers (with retention times of 5.03 and 5.63 min) were detected in *S. reticulata*, whereas procyanidin trimer T was determined in *P. niruri*. On the other hand, flavan-3-ols monomers, namely (+)-catechin and (−)-epicatechin, were found in both *P. niruri* and *S. reticulata*, constituting to our knowledge, the first report for both monomers in this latter plant species. In contrast, proanthocyanidins were not detected in *P. alliaceae* extract.

However, *P. alliacea* was the richest extract in phenolic acids, particularly in hydroxybenzoic acids which constituted 82.9% of total phenolic content whereas hydroxycinnamic acids accounted for 17.1% of such content. *S. reticulata* exhibited the highest proportion of hydroxycinnamic acids (65.5%), followed by hydroxybenzoic acids (30.5%) and proanthocyanidin monomers (i.e., (+)-catechin and (−)-epicatechin) (2.5%). *S. reticulata* also showed two propelargonidin dimers (1.5%), which constitute an important group of compounds due to their particular bioactivities [6,19]. Finally, *P. niruri* presented the highest content of proanthocyanidins, representing 41.6% of total phenolic content, hydroxybenzoic acids representing the remaining 54.6% of the total content.

*P. niruri* also exhibited the highest structural diversity of proanthocyanidins. Besides the flavan-3-ol monomers (−)-epicatechin and (+)-catechin constituting 17.4% and 9.8%, respectively, procyanidin dimers accounted for 13.1% and procyanidin trimer T2 [(−)-epicatechin-(4β→8)-(−)-epicatechin-(4β→8)-(+)-catechin] for 1.4%. These oligomers are important compounds because of their bioactivity as anti-inflammatory agents [20].

Procyanidin dimer B4 [(+)-(catechin-(4α→8)-(−)-epicatechin] (3.9%) was the most abundant in the extract of *P. niruri*, followed by B2 [(−)-epicatechin-(4α→8)-(−)-epicatechin] (3.8%), B3 [(+)-catechin-(4α→8)-(+)-catechin] (2.4%) and B1 [(−)-epicatechin-(4β→8)-(+)-catechin] (2.3%), while procyanidin B5 [(−)-epicatechin-(4β→6)-(−)-epicatechin] showed the lowest proportion (0.7%).

Regarding phenolic acids, salicylic acid (37.2%) and benzoic acid (33.5%) were the main components in *P. alliaceae*, followed by ferulic acid (10%), p-coumaric acid (6.7%) and 4-hydroxybenzoic acid (5.9%). In *P. niruri* extract, gallic acid is the single most abundant phenolic acid (40.0%), followed by protocatechuic acid (10.1%); whereas in *S. reticulata*, ferulic acid is the main component (52.6%) followed by 4-hydroxybenzoic acid (11.4%), caffeic acid (7.4%), vanillic acid (7.0%), p-coumaric acid (5.5%) and protocatechuic acid (5.1%).

The total UPLC contents were in agreement with the total phenolics (TPC) as measured by the Folin-Ciocalteau (Table 1 and Table 2). For instance, *P. niruri* showed the higher contents by both methods (TPC of 328.8 mg GAE/g extract and UPLC value of 1909.9 µg/g extract), followed by *S. reticulata* which exhibited intermediate values (TPC of 72.3 mg GAE/g extract and UPLC value of 708.8 µg/g extract), and finally by *P. alliacea* (TPC of 13.45 mg GAE/g extract and UPLC value of 473.0 µg/g extract).

Finally, the total proanthocyanidin (PRO) contents was also in agreement with the UPLC findings. For instance, *P. niruri* showed the highest PRO value by both methods (322.93 mg CCE/g extract and UPLC value of 794.5 µg/g extract), followed by *S. reticulata* (PRO of 22.35 mg CCE/g extract and UPLC value of 25.6 µg/g extract), whereas no proanthocyanidin content were obtained by either method for *P. alliaceae.*

### 2.3. Antioxidant Activity

The DPPH and ORAC values are summarized in Table 3. In both antioxidant tests, values varied in the following order: *P. niruri* (IC_50_ (DPPH) 6.4 μg/mL and 6.5 mmol Trolox equivalents/g) > *S. reticulata* (IC_50_ (DPPH) 72.9 μg/mL and 2.68 mmol Trolox equivalents/mg) > *P. alliaceae* (IC_50_ (DPPH) > 1000 μg/mL and 1.32 mmol Trolox eq/mg).

Regarding antioxidant values, our results are in agreement with other published results. For instance, low antioxidant values have also been reported for hydro-alcoholic extracts of *P. alliaceae* (DPPH, IC_50_ 255 µg/mL) [21]. For *S. reticulata*, values varied according to the method (TEAC, 0.03 mmoL TE/g; ORAC, 0.23 mmol TE/g) [17] and were lower when compared to ORAC values obtained herein. There is no report on DPPH for *S. reticulata*, but when comparing to other *Senna* species, IC_50_ values range between 89–424 µg/mL for ethanolic extracts of *S.gardneri*, *S. splendida*, *S.macranthera* and *S. trachypus* [22], thus a better result is obtained in our case for *S. reticulata* enriched-extract.

Finally, the higher antioxidant values found for *P. niruri* are in agreement with reports from the literature using DPPH method. For instance, studies on aqueous extracts report IC_50_ values of 15.3 µg/mL [3] and 6.85 µg/mL [18], whereas IC_50_ values of 9.1 µg/mL and 11.07 µg/mL were reported for methanolic [3] and ethanolic [18] extracts respectively. However, other studies reported lower antioxidant activity for a hydro-alcoholic extract (IC_50_ = 32.64 µg/mL) [10]. Therefore, our DPPH results for *P. niruri* are better than previous studies.

The difference in antioxidant values among *P. alliaceae*, *S. reticulata* and *P. niruri* extracts could be attributed to the differences in their phenolic content and distribution. Therefore, in order to investigate if the phenolic composition contributes to the antioxidant activity, a correlation analysis was carried out between DPPH and ORAC values, and the total phenolic contents (TPC, Table 1), as well as with the content by UPLC (Table 3). No correlation was found for DPPH, however, a significant and positive correlation was observed between TPC and ORAC values (*R*^2^ = 0.996) and between UPLC contents and ORAC values (*R*^2^ = 0.990). Therefore, our results are in agreement with previous studies reporting correlation between total polyphenolic contents and ORAC antioxidant activity [17].

### 2.4. Cytotoxicity of P. alliaceae, P. niruri and S. reticulata Extracts 

**Figure 2:**
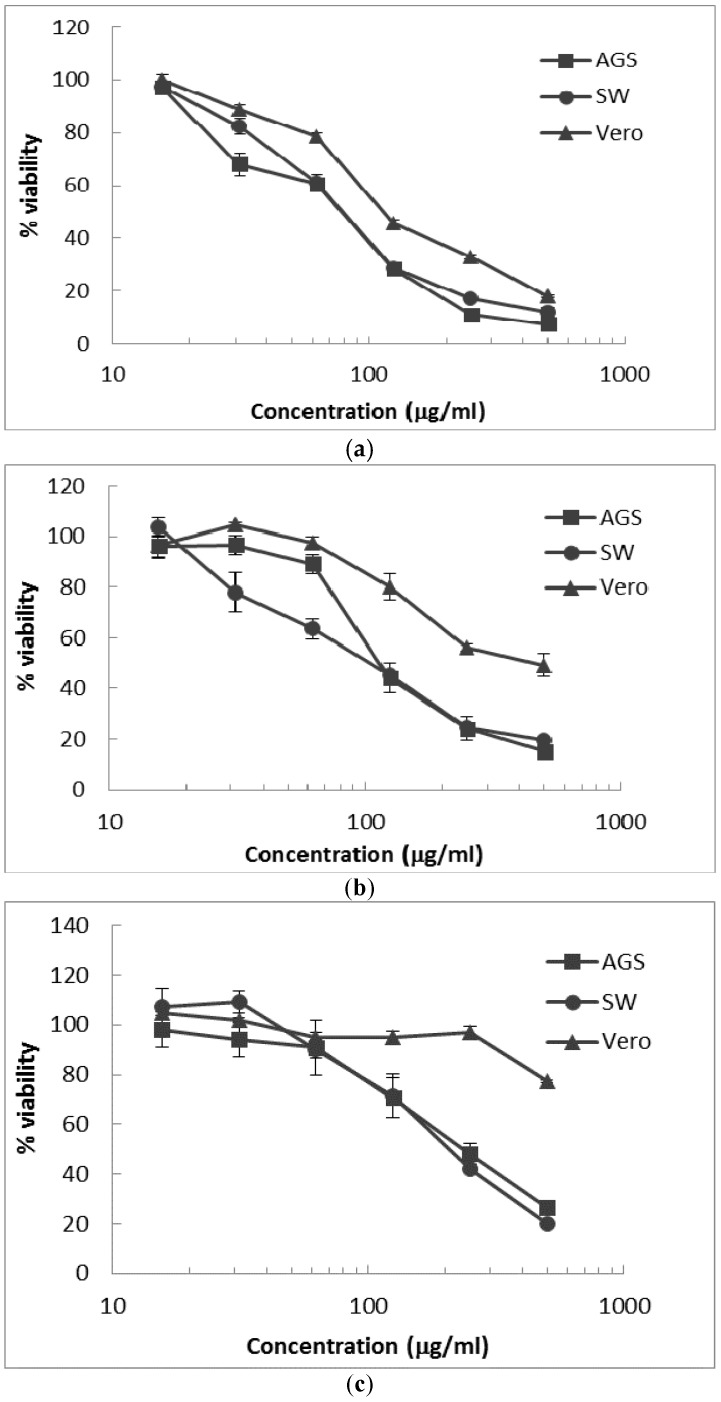
Cytotoxicity dose-response curves of extract treatment on tumoral cell lines. (**a**) *Petiveria alliacea*, (**b**) *P. niruri*, (**c**) *S. reticulate*. Results represent the mean ± SE of triplicates of one representative experiment of each cell line.

Table 4 summarizes the IC_50_ values for the cytotoxicity of *P. alliaceae*, *P. niruri* and *S. reticulata* extracts on AGS human gastric adenocarcinoma, SW620 human colon adenocarcinoma and Vero monkey normal epithelial kidney cell lines. Also, Figure 2 shows dose-response curves. IC_50_ values indicate that there is no significant difference (one-way analysis of variance (ANOVA) followed by Tukey’s post hoc test) between cytotoxicity values (*p* < 0.05) against gastric AGS adenocarcinoma cells and SW620 adenocarcinoma cells for *P. alliacea* and *S. reticulata* extracts. However, in the case of *P. niruri* extract, the ANOVA indicates that the cytotoxicity results are dependent on the cancer cell line.

Our results for the cytotoxicity of *P. alliacea* on both adenocarcinoma cell lines are similar to those reported for an ethanolic extract on Jurkat T cells [23]. Other studies indicate variability of results, with IC_50_ values ranging from 29 to 36 µg/mL for a C-18 chromatographic fraction of a hydro-alcoholic extract on erythroleukema and melanoma cell lines [24], and breast adenocarcinoma 4T1 cell line [25]. In contrast, no cytotoxic effect was observed for a methanolic extract on hepatic adenocarcinoma HepG2 [26].

For *S. reticulata* extracts, the results for the cytotoxicity are in agreement with a study reporting IC_50_ of 232.9 µg/mL for a methanolic extract on KB nasopharyngeal cells. However, no cytotoxicity was found in aqueous extracts [27]. Studies on other species of the genus *Senna*, including a methanolic extract of *S. covesii*, also reported no activity (IC_50_ > 800 µg/mL) on L929 tumor connective tissue cell line, HeLa cervix carcinoma and C3F6 lymphoma [28].

Finally, the cytotoxicity results obtained for the *P. niruri* extract on AGS tumor gastric cell lines and SW620 tumor colon cell lines, are similar to those found in the literature for methanolic and aqueous extracts on PC-3 prostate cancer cell lines (IC_50_ values of 117.7 and 155.0 µg/mL respectively), MeWo skin cancer cell lines (IC_50_ values of 153.3 and 193.3 µg/mL respectively) [29], and for an hydro-methanolic extract on MCF-7 human breast carcinoma cell lines (IC_50_ of 84.88 µg/mL) [30]. Similar to our cell line-dependence findings for *P. niruri* phenolic extracts, other studies performed on hydro-methanolic extracts of *P. amarus* and *P. virgatus* report lower cytotoxicity on Hep G2 hepatic carcinoma (IC_50_ > 250 µg/mL) [31].

Concerning the selectivity of the cytotoxic activity of extracts against cancerous cell in normal Vero cells, our results indicate significant difference (ANOVA, *p* < 0.05) between IC_50_ values for both AGS and SW620 adenocarcinoma cell lines and normal Vero cells. When comparing selectivity index (SI), defined as the ratio of IC_50_ values of normal (Vero) cells to cancer cells (AGS or SW620), *P. alliaceae* extract showed the lowest values on both cell lines (SI = 1.4), while *P. niruri* extract showed the best selectivity result for SW620 colon cancer cells (SI = 2.8), which is in agreement with the selectivity range (SI ≥ 3) reported as promising for further anticancer studies [32,33]. In turn, *S. reticulata* extract exhibited a slightly lower value on SW620 colon cancer cells (SI = 2.5) and on AGS gastric cancer cells (SI = 2.4), followed by *P. niruri* extract on SW620 cells (SI = 2.2). This variability has been reported in previous studies, showing selective cytotoxicity of a polyphenolic extract between gastric normal and cancer cells [34], whereas other studies report no selectivity on the cytotoxic effect in normal, as well as in cancer breast and prostate cell lines [35].

In fact, mechanisms related to the effect of polyphenols in cancer cells need to be elucidated, taking into account, for instance, the multiplicity of targets that can be reached [14] and the complexity of factors modulating cancer cell phenotypes [36]. However, there is enough evidence to support the potential anticancer effects of proanthocyanidins [12]. For instance, previous studies using proanthocyanidins from grape seeds showed a cytotoxic effect in cervix cancer [37], whereas cranberries polyphenols inhibited proliferation in prostate and colon cancer cells [38]. Also, other studies indicated the potential of propelargonidin dimers and procyanidin dimers and contents on higher and more selective cytotoxicity in gastric and colon cancer cell lines [6]. In addition, a report on another main component of *P. niruri* extract, gallic acid, indicates that this compound inhibits the growth of human hepatocellular carcinoma cells and induces apoptosis in these cell lines [39]. Since polyphenols could work in a synergistic manner, fractioning of *S. reticulata* and *P. niruri* extracts would contribute to further elucidate the structure-bioactivity relationship of these plant extracts.

## 3. Materials and Methods

### 3.1. Materials, Reagents and Solvents

*Petiveria alliaceae*, *Phyllanthus niruri* and *Senna reticulata* aerial plant material were acquired from local communities that grow and use the plants as traditional medicine, grouped as an Agricultural Producers Association (AMPALEC) in the Caribbean region. All plants were identified with the support of the Costa Rican National Herbarium and vouchers are deposited there. The material of each plant was rinsed in water and cut into small pieces. Subsequently, it was dried in a stove at 40°C until completely dry, and after being ground, it was preserved at −5 °C. Reagents such as AAPH, fluorescein, DPPH, and Trypsin-EDTA solution were provided by Sigma-Aldrich (St. Louis, MO, USA), while amphotericin B, penicillin-streptomycin, and Minimum essential Eagle’s medium (MEM, 10% fetal bovine serum), were purchased from Life Technologies (Carlsbad, CA, USA). AGS human gastric adenocarcinoma, SW 620 human colorectal adenocarcinoma and Vero monkey normal epithelial kidney cell lines were obtained from American Type Culture Collection (ATCC, Rockville, MD, USA). DMSO was provided by Sigma-Aldrich (St. Louis, MO, USA), while MTBE, chloroform and methanol were purchased from Baker (Center Valley, PA, USA).

### 3.2. Phenolic Extracts from P. alliaceae, P. niruri and S. reticulata

The process followed for obtaining phenolic-enriched extracts was formerly described by our group [40]. Briefly, dried material from each plant was first extracted in a mixture of methyl ter-butyl ether (MTBE) and methanol (MeOH) 90:10 (*v*/*v*) at 25 °C during 30 min in ultrasound. Afterwards it was extracted for 24 h in order to obtain a non-polar extract of the samples. After filtration, the extraction was repeated once. The extracts were combined and the solvents evaporated in a rotavapor to dryness and subsequently washed with MeOH in order to extract any polyphenols. The residual plant material was extracted with MeOH at 25 °C during 30 min in ultrasound, and then extracted for 24 h. After filtration, the extraction was repeated twice. The three methanol extracts were combined with the previous MeOH washings and were evaporated in a rotavapor to dryness. Finally, the dried extract was washed with hexane, MTBE and chloroform consecutively in order to obtain a phenolic rich-extract.

### 3.3. Total Phenolic Content

The polyphenolic content was determined by a modification of the Folin-Ciocalteu (FC) method [41], whose reagent is composed of a mixture of phosphotungstic and phosphomolybdic acids. Each sample was dissolved in MeOH (0.1% HCl) and combined with 0.5 mL of FC reagent. Afterwards 10 mL of Na_2_CO_3_ (7.5%) were added and the volume was completed to 25 mL with water. Blanks were prepared in a similar way but using 0.5 mL of MeOH (0.1% HCl) instead of sample. The mixture was let standing in the dark for 1 h and then absorbance was measured at 750 nm. Values obtained were extrapolated in a gallic acid calibration curve. Total phenolic content was expressed as mg gallic acid equivalents (GAE)/g sample. Analyses were performed in triplicate.

### 3.4. Total Proanthocyanidin Content

The proanthocyanidin content was determined by a modification of the Bate-Smith method, which consists of the cleavage of the C–C interflavanic bond of proanthocyanidins in butanol-HCl through oxidative acid-catalysis [42]. Briefly, 0.2 mL of each sample were mixed with 20 mL of butanol/HCl (50:50) and 0.54 mM FeSO_4_. The mixture was incubated at 90 °C for 1 h and after cooling, the volume was completed to 25 mL with butanol-HCl mixture. The absorbance was measured at 550 nm against a blank prepared in a similar way but without heating. The standard used was cyanidin chloride (which served to draw a calibration curve. Results were expressed as mg of cyanidin chloride equivalents (CAE)/g of extract.

### 3.5. UPLC-DAD-ESI-TQ-MS Analysis

The UPLC-MS system used to analyze the phenolic composition of the samples consisted of an UPLC coupled to an Acquity PDA eλ photodiode array detector (DAD) and an Acquity TQD tandem quadrupole mass spectrometer equipped with Z-spray electrospray interface (UPLC-DAD-ESI-TQ-MS) (Waters, Milford, MA, USA). The analyses were performed using a solution of 5 mg/mL of each extract in acetonitrile:H_2_O (1:4). A volume of 2 μL was injected and a Waters^®^ BEH C18 column (2.1 × 100 mm; 1.7 µm) was used. The elution consisted of a gradient composed of solvent A-water:acetic acid (98:2, *v*/*v*) and B-acetonitrile:acetic acid (98:2, *v*/*v*) [43]: 0–1.5 min: 0.1% B, 1.5–11.17 min: 0.1–16.3% B, 11.17–11.5 min: 16.3–18.4% B, 11.5–14 min: 18.4% B, 14–14.1 min: 18.4–99.9% B, 14.1–15.5 min: 99.9% B, 15.5–15.6 min: 0.1% B, 15.6–18 min: 0.1% B. The flow rate was 0.5 mL/min and DAD was recorded between 250–420 nm. ESI negative mode parameters included: source temperature, 130 °C; capillary voltage, 3 kV; desolvation temperature, 400 °C; cone gas (N_2_) flow rate, 60 L/h; and desolvation gas (N_2_) flow rate, 750 L/h. For quantification, MRM transitions were used, such as *m*/*z* 169/125 for gallic acid, *m*/*z* 289/245 for (+)-catechin and (−)-epicatechin, *m*/*z* 577/289 for procyanidin dimers, *m*/*z* 561/289 for propelargonidin dimers, and m/z 865/577 for procyanidin trimers. Commercial standards used were (−)-epicatechin, (+)-catechin, procyanidins B1 and B2. Assignment of procyanidins B3, B4 and B5 and procyanidin trimer T2 was performed with previously isolated standards and confirmed by MS/MS spectrum. Assignment of propelargonidins was performed through MS/MS spectrum at m/z 561 and quantification was performed on the calibration curve of procyanidin B1. The limit of detection (LOD) and limit of quantification (LOQ) are published elsewhere [43,44].

### 3.6. DPPH Radical-Scavenging Activity

A solution of 2,2-diphenyl-1-picrylhidrazyl (DPPH) (0.25 mM) was prepared using methanol as solvent. Next, 0.5 mL of this solution were mixed with 1 mL of extract at different concentrations, and incubated at 25° C in the dark for 30 min. DPPH absorbance was measured at 517 nm. Blanks were prepared for each concentration. The percentage of the radical-scavenging activity of the sample was plotted against its concentration to calculate IC_50_, which is the amount of sample required to reach the 50% radical-scavenging activity. The samples were analyzed in triplicate.

### 3.7. ORAC Antioxidant Activity

Extracts (0.05 g) were mixed with 10 mL of methanol/HCl (1000:1, *v*/*v*) and sonicated for 5 min. Afterwards, the mixture was centrifuged and filtered. Fluorescein was used as fluorescence probe [45]. The reaction was performed in 75 mM phosphate buffer (pH 7.4) at 37 °C. The final assay mixture consisted of AAPH (12 mM), fluorescein (70 nM), and either Trolox (1–8 µM) or the extract at different concentrations. Fluorescence was recorded every minute for 98 min in black 96-well untreated microplates (Nunc, Denmark), using a Polarstar Galaxy plate reader (BMG Labtechnologies GmbH, Offenburg, Germany) with 485-P excitation and 520-P emission filters. Fluostar Galaxy software version 4.11-0 (BMG Labtechnologies GmbH, Offenburg, Germany) was used to measure fluorescence. Fluorescein was diluted from a stock solution (1.17 mM) in 75 mM phosphate buffer (pH 7.4), while AAPH and Trolox solutions were freshly prepared. All reaction mixtures were prepared in duplicate and three independent runs were completed for each extract. Fluorescence values obtained were normalized to the curve of the blank (no antioxidant). The area under the fluorescence decay curve (AUC) was calculated from the normalized curves, and the net AUC was then established. Subsequently, regression equation between antioxidant concentration and net AUC was obtained. Finally, ORAC value was estimated by dividing the slope of the latter equation by the slope of the Trolox line. ORAC values were expressed as mmol of Trolox equivalents (TE)/g of extract.

### 3.8. Evaluation of Cytotoxicity

The AGS, SW620 and Vero cells were grown in MEM (10% FBS) in the presence of glutamine (2 mmol/L), penicillin (100 IU/mL), streptomycin (100 µg/L) and amphotericin B (0.25 µg/m), at 37 °C, in a humidified atmosphere (5% CO_2_) [5]. Briefly, 100 µL of 1.5 × 10^5^ cells/mL (suspension) were seeded overnight into 96-well plates to reach 100% confluency. Subsequently, the cells were exposed for 48 h to 50 µL of extracts in concentrations varying 15–500 µg/mL in MEM (DMSO 0.1% *v*/*v*). Afterwards, the medium was eliminated, cells were washed with PBS (100 µL) and incubated with 100 µL of a MTT solution (0.5 mg/mL, final concentration) in PBS, for 2 h at 37 °C. Then, MTT was removed and the formazan crystals were dissolved in 100 µL of ethanol 95%. Absorbance was read at 570 nm in a microplate reader. DMSO was diluted in media in the same way as the extracts and incubated with the cells for 48 h to be used as control. Dose-response curves were established and IC_50_ was calculated. Extracts were tested in three independent experiments with different doses of extract analyzed in triplicate.

## 4. Conclusions

This study represents the first detailed MS analysis of phenolic-enriched extracts of *P. alliaceae, P. niruri and S. reticulata*, three species commonly used in traditional medicine in Costa Rica. Using different methods, including UPLC-DAD-ESI-TQ-MS techniques, results show diverse contents and distribution of 20 phenolic acids and proanthocyanidins among extracts. These findings constitute the first report on the diversity of phenolic acids in *P. alliaceae* and *S. reticulata*, and the presence of proanthocyanidins as minor components in this latter extract. In addition, five procyanidin dimers and one procyanidin trimer, were also detected for the first time in *P. niruri*. Further, significant positive correlation was found between total phenolic contents (TPC) and ORAC (*R*^2^ = 0.996) antioxidant value as well as between UPLC contents and ORAC (*R*^2^ = 0.990). *P. niruri* extract showed the highest antioxidant values in both DPPH and ORAC methods, as well as better cytotoxicity and selectivity on AGS gastric adenocarcinoma and SW620 colon adenocarcinoma cell lines in respect to normal cells. These results suggest that the high content of proanthocyanidins (41.6% of total phenolic content) found in *P. niruri* extracts could be responsible for the higher cytotoxicity and selectivity of the plant compared to the other two species from this study. Finally, the results show evidence of the potential health effects of *P. niruri* extracts on gut-related diseases, considering these polyphenols are metabolized by the gut [15,43]. Purification or fractioning of *P. niruri* phenolic extracts would be of interest to further evaluate their structure-bioactivity relationship.

## Figures and Tables

**Table 1:** Extraction yield and total phenolic content.

Sample	Extraction Yield (%) ^1^	Total Phenolic Content (TPC) (mg/g) ^2,5^	Total Proanthocyanidin Contents (PRO) (mg/g) ^3,5^
*P. alliacea*	5.03	13.45 ^a^ ± 0.46	nd ^4^
*P. niruri*	5.58	328.80 ^b^ ± 13.41	322.93 ^a^ ± 11.12
*S. reticulata*	6.53	79.30 ^c^ ± 4.09	22.35 ^b^ ± 1.64

^1^ g of extract/g of dry material expressed as %. ^2^ mg of gallic acid equivalent (GAE)/g extract. ^3^ mg of cyanidin chloride equivalent (CCE)/g extract. ^4^ nd = not detected. ^5^ Different superscript letters in the same column indicate differences are significant at *p* < 0.05.

**Table 2:** Phenolic composition of *P. alliaceae*, *P. niruri* and *S. reticulata* extracts.

Compounds	*P. alliaceae*	*P. niruri*	*S. reticulata*
	Concentration (µg/g Extract)		
*Hydroxybenzoic acids*			
Benzoic acid	158.4 ± 7.5	nd	nd
Salicylic acid	175.9 ± 2.4	61.2 ± 1.3	16.7 ± 0.1
4-Hydroxybenzoic acid	28.1 ± 0.2	14.3 ± 0.1	80.9 ± 1.2
Protocatechuic acid	6.3 ± 0.2	192.4 ± 0.9	36.3 ± 0.3
Gallic acid	2.4 ± 0.0	763.3 ± 8.1	7.5 ± 0.3
Vanillic acid	12.1 ± 0.1	10.7 ± 0.2	49.6 ± 1.7
Syringic acid	9.2 ± 0.4	nd	25.0 ± 0.8
*∑ Hydroxybenzoic acids*	392.4	1041.9	216.0
*Hydroxycinnamic acids*			
*p*-Coumaric acid	31.6 ± 0.4	13.5 ± 0.8	39.0 ± 0.9
Caffeic acid	1.6 ± 0.0	25.0 ± 0.8	52.8 ± 0.6
Ferulic acid	47.5 ± 1.1	34.7 ± 1.1	372.5 ± 9.5
*∑ Hydroxycinnamic acids*	80.7	73.2	464.3
*Flavan-3-ols: monomers*			
(+)-Catechin	nd	186.6 ± 6.4	3.7 ± 0.1
(−)-Epicatechin	nd	331.7 ± 8.3	14.0 ± 0.1
*∑ Monomers*	nd	518.3	17.7
*Flavan-3-ols: procyanidin dimers*			
Procyanidin B1	nd	44.2 ± 1.5	nd
Procyanidin B2	nd	73.0 ± 3.2	nd
Procyanidin B3	nd	45.8 ± 1.6	nd
Procyanidin B4	nd	74.0 ± 1.5	nd
Procyanidin B5	nd	13.2 ± 0.3	nd
*∑ Procyanidin dimers*	nd	250.2	nd
*Flavan-3-ols: propelargonidin dimers*			
Propelargonidin dimer (5.03 min)	nd	nd	4.9 ± 0.1
Propelargonidin dimer (5.63 min)	nd	nd	5.9 ± 0.2
*∑ Properlargonidin dimers*	nd	nd	10.8
*Flavan-3-ols: procyanidin trimers*			
Trimer T2	*nd*	26.0 ± 0.6	nd
*∑ Procyanidin trimers*	*nd*	26.0	nd

nd—not detected.

**Table 3:** Total phenolic contents (UPLC-DAD-ESI-TQ-MS analysis) and antioxidant activity.

Sample	Total Phenolics UPLC ^1^ (µg/g Extract)	DPPH ^2^ IC50 (μg/mL)	ORAC ^2^ (mmol TE/mg Extract)
*P. alliacea*	473.0	>1000 ^a^	1.32 ^a^ ± 0.11
*P. niruri*	1909.6	6.40 ^b^ ± 0.10	6.50 ^b^ ± 0.15
*S. reticulata*	708.8	72.90 ^c^ ± 1.10	2.68 ^c^ ± 0.28

^1^ Σ = [hydroxybenzoic acids + hydroxycinnamic acids + flavan-3-ols monomers + procyanidin dimers + propelargonidin dimers + procyanidin trimers] contents (µg/g extract) (Table 2). ^2^ Different superscript letters in the same column indicate differences are significant at *p* < 0.05.

**Table 4:** Cytotoxicity of extracts to gastric (AGS) and colon (SW620) adenocarcinoma cells as well as to control Vero cells.

Sample	IC_50_ (µg/mL)		
	AGS ^1^	SW620 ^1^	Vero ^1^
*P. alliacea* ^2^	106.5 ^a,^* ± 7.9 (SI = 1.4)	108.4 ^a,^* ± 4.7 (SI = 1.4)	151.5 ^a,+^ ± 3.3
*P. niruri* ^2^	145.2 ^b,^* ± 8.2 (SI = 2.2)	113.2 ^a,+^ ± 4.3 (SI = 2.8)	311.9 ^b,^^◊^ ± 24
*S. reticulata* ^2^	208.4 ^c,^* ± 8.9 (SI = 2.4)	202.5 ^b,^* ± 9.1 (SI = 2.5)	>500 ^c,+^

^1^ Different superscript letters in the same column indicate differences are significant at *p* < 0.05. ^2^ Different superscript signs in the same row indicate differences are significant at *p* < 0.05.

## Supplementary Materials

The following are available online at http://www.mdpi.com/2223-7747/6/4/50/s1, Figure S1: Chromatograms (UPLC-DAD) for polyphenolic compounds: (**a**) *P. alliaceae* extract, (**b**) *P. niruri* extract, (**c**) *S. reticulata* extract, Table S1: UPLC and MS/MS parameters for the identified polyphenols.

## References

[1] Arnaez E., Moreira I., Navarro M. (2016). Manejo Agroecológico de Nueve Especies de Plantas de uso Tradicional Cultivadas en Costa Rica.

[2] Nunes dos Santos R., Vasconcelos Silva M.G. (2008). Constituintes químicos do caule de *Senna reticulata* Willd. (Leguminoseae). Quim. Nova.

[3] Harish R., Shivanandappa T. (2006). Antioxidant activity and hepatoprotective potential of *Phyllanthus niruri*. Food Chem..

[4] Kim S., Kubec R., Musah R.A. (2006). Antibacterial and antifungal activity of sulfur-containing compounds from *Petiveria alliacea* L.. J. Ethnopharmacol..

[5] Navarro-Hoyos M., Lebrón-Aguilar R., Quintanilla-López J.E., Cueva C., Hevia D., Quesada S., Gabriela Azofeifa G., Moreno-Arribas M.V., Monagas M., Bartolomé B. (2017). Proanthocyanidin Characterization and Bioactivity of Extracts from Different Parts of *Uncaria tomentosa* L. (Cat’s Claw). Antioxidants.

[6] Navarro M., Zamora W., Quesada S., Azofeifa G., Alvarado D., Monagas M. (2017). Fractioning of Proanthocyanidins of *Uncaria tomentosa*. Composition and Structure-Bioactivity Relationship. Antioxidants.

[7] Demirezer L.O., Karahan N., Ucakturk E., Kuruuzum-Uz A., Guvenalp Z., Kazaz C. (2011). HPLC Fingerprinting of sennosides in laxative drugs with isolation of standard substances from some *Senna* Leaves. Rec. Nat. Prod..

[8] Araújo-Luz D., Miranda-Pinheiro A., Lopes-Silva M., Chagas-Monteiro M., Prediger R.D., Ferraz-Maia C.S., Andrade-Fontes E. (2016). Ethnobotany, phytochemistry and neuropharmacological effects of *Petiveria alliacea* L. (Phytolaccaceae): A review. J. Ethnopharmacol..

[9] Bagalkotkar G., Sagineedu S.R., Saad M.S., Stanslas J. (2006). Phytochemicals from *Phyllanthus niruri* Linn. and their pharmacological properties: A review. J. Pharm. Pharmacol..

[10] Mahdi E., Noor A., Sakeena M., Abdullah G., Abdulkarim M., Sattar M. (2011). Identification of phenolic compounds and assessment of in vitro antioxidants activity of 30% ethanolic extracts derived from two *Phyllanthus* species indigenous to Malaysia. J. Pharm. Pharmacol..

[11] Mediani A., Abas F., Khatib A., Tan C.P., Ismail I.S., Shaari K., Ismail A., Lajis N.H. (2015). Relationship between metabolites composition and biological activities of *Phyllanthus niruri* extracts prepared by different drying methods and solvents extraction. Plant Foods Hum. Nut..

[12] Zhou K., Raffoul J.J. (2012). Potential anticancer properties of grape antioxidants. J. Oncol..

[13] Considine M.J., Foyer C.H. (2014). Redox Regulation of Plant Development. Antioxidants & Redox signaling. Antioxid. Redox Signal..

[14] Barrajon-Catalan E., Herranz-López M., Joven J., Segura-Carretero A., Alonso-Villaverde C., Menéndez J.A., Micol V., Camps J. (2014). Oxidative Stress and Inflammation in Non-Communicable Diseases—Molecular Mechanisms and Perspectives in Therapeutics.

[15] Monagas M., Urpi-Sarda M., Sanchez-Patán F., Llorach R., Garrido I., Gómez-Cordoves C., Andres-Lacueva C., Bartolome B. (2010). Insights into the metabolism and microbial biotransformation of dietary flavan-3-ols and the bioactivity of their metabolites. Food Funct..

[16] Zaa C., Valdivia M., Marcelo A. (2012). The anti-inflammatory and antioxidant effect of hydroalcoholic extract of *Petiveria alliacea*. Rev. Peru. Biol..

[17] Lizcano L.J., Bakkali F., Ruiz-Larrea B., Ruiz-Sanz J.I. (2010). Antioxidant activity and polyphenol content of aqueous extracts from Colombia Amazonian plants with medicinal use. Food Chem..

[18] Amin Z.A., Abdulla M.A., Ali H.M., Alshawsh M.A., Qadir S.W. (2012). Assessment of In vitro antioxidant, antibacterial and immune activation potentials of aqueous and ethanol extracts of *Phyllanthus niruri*. J. Sci. Food Agric..

[19] Chang E., Lee W., Cho S., Choi S. (2003). Proliferative effects of Flavan3-ols and Propelargonidins from Rhizomes of *Drynaria fortune* on MCF-7 and Osteoblastic Cells. Arch. Pharm. Res..

[20] Montagut G., Baiges I., Valls J., Terra X., Bas J., Vitrac X., Richard T., Mérillon J., Arola L., Blay M. (2009). A trimer plus a dimer-gallate reproduce the bioactivity described for an extract of grape see procyanidins. Food Chem..

[21] Ramos A., Visozo A., Piloto J., García A., Rodríguez C.A., Rivero R. (2003). Screening of antimutagenicity via antioxidant activity in Cuban medicinal plants. J. Etnopharmacol..

[22] Silva G.A., Monteiro J.A., Ferreira E.B., Fernandes M.I.B., Pessoa C., Sampaio C.G., Silva M.G.V. (2014). Total phenolic content, antioxidant and anticancer activities of four species of *Senna* Mill. From northeast Brazil. Int. J. Pharm. Pharm. Sci..

[23] Schmidt C., Fronza M., Goettert M., Geller F., Luik S., Flores E.M.M., Bittencourt C.F., Zanetti G.D., Heinzmann B.M., Laufer S., Merfort I. (2009). Biological studies on Brazilian plants used in wound healing. J. Etnopharmacol..

[24] Urueña C., Cifuentes C., Castañeda D., Arango A., Kaur P., Asea A., Fiorentino S. (2008). *Petiveria alliacea* extracts uses multiple mechanisms to inhibit growth of human and mouse tumor cells. BMC Complement. Altern. Med..

[25] Hernandez J.F., Urueña C.P., Cifuentes M.C., Sandoval T.A., Fiorentino S. (2014). A *Petiveria alliacea* standardized fraction induces breast adenocarcinoma cell death modulating glycolytic metabolism. J. Ethnopharmacol..

[26] Ruffa M.J., Ferrar G., Wagner M.L., Calcagno M.L., Campos R.H., Cavallaro L. (2002). Cytotoxic effect of argentine medicinal plant extracts on human hepatocellular carcinoma cell line. J. Etnopharmacol..

[27] Camacho M.D., Phillipson J.D., Croft S.L., Solis P.N., Marshall S.J., Ghazanfar S.A. (2003). Screening of plants extracts for antiprotozoal and cytotoxic activities. J. Ethnopharmacol..

[28] Jiménez-Estrada M., Velásquez-Contreras C., Garibay-Escobar A., Sierras-Canchola D., Lapisco-Vásquez R., Ortiz-Sandoval C., Burgos-Hernández A., Robles-Zepeda R. (2013). In vitro antioxidant and antiproliferative activities of plants of the ethnopharmacopeia from northwest of Mexico. BMC Complement. Altern. Med..

[29] Tang Y.Q., Jaganath I.B., Sekaran S.D. (2010). *Phyllanthus* spp. induces selective growth inhibition of PC-3 and MeWo human cancer cells through modulation of cell cycle and induction of apoptosis. PLoS ONE.

[30] Jose J., Sudhakaran S., Sumesh-Kumar T.M., Jayadevi-Variyar E., Jayaraman S. (2014). A comparative evaluation of anticancer activities of flavonoids isolated from *Mimosa pudica, Aloe vera* and *Phyllanthus niruri* against human breast carcinoma cell line (MCF-7) using MTT assay. Int. J. Pharm. Pharm. Sci..

[31] Poompachee K., Chudapongse N. (2012). Comparison of the antioxidant and cytotoxic activities of *Phyllanthus virgatus* and *Phyllanthus amarus* extracts. Med. Princ. Pract..

[32] Mahavorasirikul W., Wiratchanee M., Vithoon V., Wanna C., Arunporn I., Kesara N. (2010). Cytotoxic activity of Thai medicinal plants against human cholangiocarcinoma, laryngeal and hepatocarcinoma cells in vitro. BMC Complement. Altern. Med..

[33] Ramasamy S., Wahab N.A., Abidin N.Z., Manickam S., Zakaria Z. (2012). Growth Inhibition of Human Gynecologic and Colon Cancer Cells by *Phyllanthus watsonii* through Apoptosis Induction. PLoS ONE.

[34] Ye X., Krohn R.L., Liu W., Joshi S.S., Kuszynski C.A., McGinn T.R., Bagchi M., Preuss H.G., Stohs S.J., Bagchi D. (1999). The cytotoxic effects of a novel IH636 grape seed proanthocyanidin extract on cultured human cancer cells. Mol. Cell. Biochem..

[35] Weaver J., Briscoe T., Hou M., Goodman C., Kata S., Ross H., McDougall G., Stewart D., Riches A. (2009). Strawberry polyphenols are equally cytotoxic to tumourigenic and normal human breast and prostate cell lines. Int. J. Oncol..

[36] Stoner G., Wang L., Casto B. (2008). Laboratory and clinical studies of cancer chemoprevention by antioxidants in berries. Carcinogenesis.

[37] Chen Q., Liu X.F., Zheng P.S. (2014). Grape seed proanthocyanidins (GSPs) inhibit the growth of cervical cancer by inducing apoptosis mediated by the mitochondrial pathway. PLoS ONE.

[38] Seeram N., Lee R., Heber D. (2004). Bioavailability of ellagic acid in human plasma after consumption of ellagitannins from pomegranate (*Punica granatum* L.) juice. Clin. Chim. Acta.

[39] Sun G., Zhang S., Xie Y., Zhang Z., Zhao W. (2016). Gallic acid as a selective anticancer agent that induces apoptosis in SMMC-7721 human hepatocellular carcinoma cells. Oncol. Lett..

[40] Navarro Hoyos M., Sánchez-Patán F., Murillo Masis R., Martín-Álvarez P.J., Zamora Ramirez W., Monagas M.J., Bartolomé B. (2015). Phenolic Assesment of *Uncaria tomentosa* L. (Cat’s Claw): Leaves, Stem, Bark and Wood Extracts. Molecules.

[41] Singleton V., Rossi J. (1965). Colorimetry of total phenolics with phosphomolybdic-phosphotungstic acid reagents. Am. J. Enol. Vitic..

[42] Ribéreau-Gayon P., Stonestreet E. (1966). Dósage des tannins du vin rouges et determination du leur structure. Chem. Anal..

[43] Sánchez-Patan F., Monagas M., Moreno-Arribas M.V., Bartolome B. (2011). Determination of microbial phenolic acids in human faeces by UPLC-ESI-TQ MS. J. Agric. Food Chem..

[44] Sánchez-Patan F., Cueva C., Monagas M., Walton G.E., Gibson G.R., Martin-Alvarez P.J., Moreno-Arribas M.V., Bartolome B. (2012). Gut microbial catabolism of grape seed flavan-3-ols by human faecal microbiota. Targetted analysis of precursor compounds, intermediate metabolites and end-products. Food Chem..

[45] Davalos A., Gomez-Cordoves C., Bartolome B. (2004). Extending applicability of the oxygen radical absorbance capacity (ORAC-Fluorescein) assay. J. Agric. Food Chem..

