# Supplementary material for: Proanthocyanidin Characterization, Antioxidant and Cytotoxic Activities of Three Plants Commonly Used in Traditional Medicine in Costa Rica: Petiveria alliaceae L., Phyllanthus niruri L. and Senna reticulata Willd
Source: Plants (Basel). 2017 Oct 19;6(4):50. doi: 10.3390/plants6040050 (PMC5750626; doi:10.3390/plants6040050)
Supplement: Supplementary file 1 [file plants-06-00050-s001.pdf]

Supplementary Material: Proanthocyanidin Characterization, Antioxidant and Cytotoxic Activities of Three Plants Commonly Used in Traditional Medicine in Costa Rica: *Petiveria alliaceae* L., *Phyllanthus niruri* L. and *Senna reticulata* Willd.

Mirtha Navarro, Ileana Moreira, Elizabeth Arnaez, Silvia Quesada, Gabriela Azofeifa, Diego Alvarado and Maria Monagas

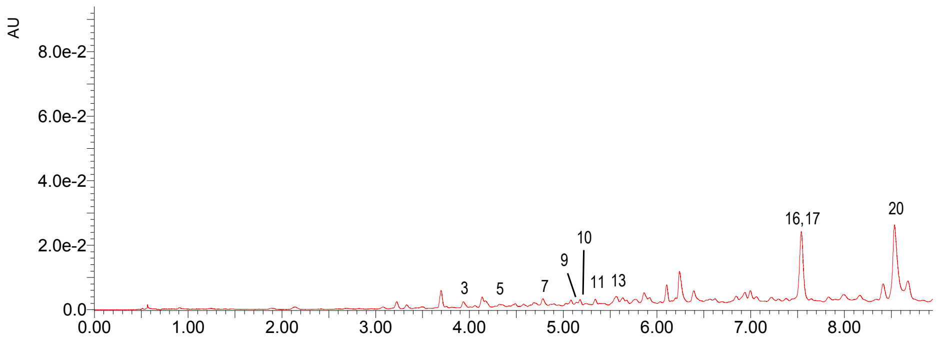

(**a**)

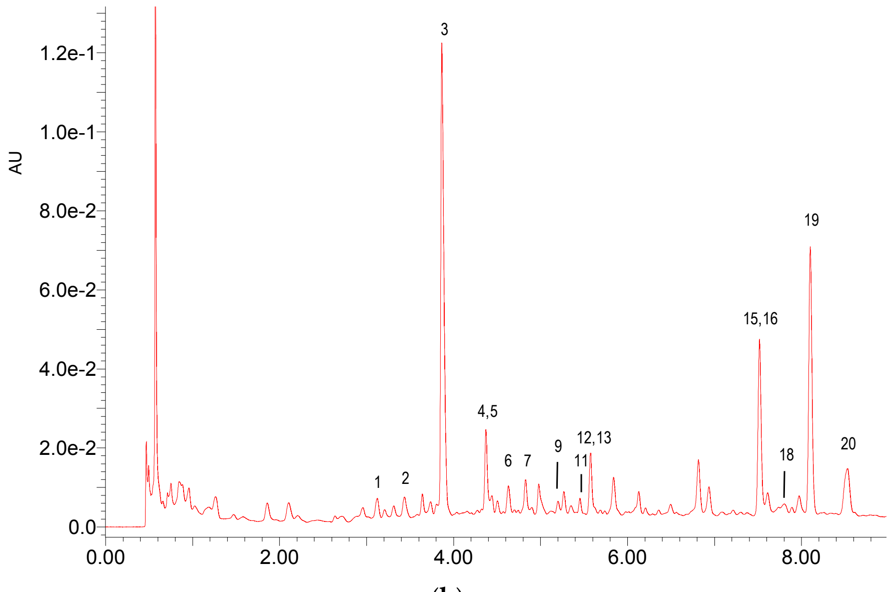

(**b**)

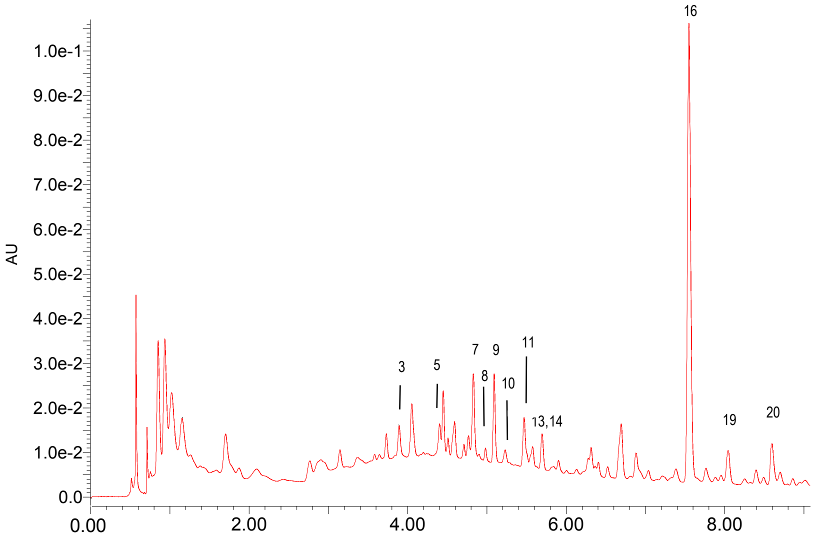

(c)

Figure S1. Chromatograms (UPLC-DAD) for polyphenolic compounds: (**a**) *P. alliaceae* extract, (**b**) *P. niruri* extract, (**c**) *S. reticulata* extract.

Table S1. UPLC and MS/MS parameters for the identified polyphenols.

#	Compuesto	Rt (min)	MRM Transition (m/z)	Collision Energy (V)	Cone Voltage (V)
1	Procyanidin B3	3.17	577 > 289	25	45
2	Procyanidin B1	3.38	577 > 289	25	45
3	Gallic acid	3.89	169 > 125	15	33
4	Procyanidin B4	4.35	577 > 289	25	45
5	Protocatechuic acid	4.37	153 > 109	12	30
6	Procyanidin B2	4.62	577 > 289	25	45
7	4-Hydroxybenzoic acid	4.79	137 > 93	12	27
8	Propelargonidin B	5.03	561 > 289	25	45
9	Caffeic acid	5.15	179 > 135	16	35
10	Syringic acid	5.20	197 > 182	12	30
11	Vanillic acid	5.41	167 > 152	12	30
12	Procyanidin T2	5.60	865 > 577	25	45
13	*p*-coumaric acid	5.59	163 > 119	14	30
14	Propelargonidin B	5.63	561 > 289	25	45
15	(+)-Catechin	7.50	289 > 245	16	40
16	Ferulic acid	7.53	193 > 134	16	30
17	Benzoic acid	7.52	121 > 77	12	27
18	Procyanidin B5	7.78	577 > 289	25	45
19	(−)-Epicatechin	8.07	289 > 245	16	40
20	Salicylic acid	8.55	137 > 93	17	27
